# Biology as a Key to Understanding the Complex Actions of Angiotensin II: An Editorial

**DOI:** 10.3390/biomedicines14102379

**Published:** 2026-10-19

**Authors:** György L. Nádasy, Mária Szekeres, László Dézsi

**Affiliations:** 1 Department of Physiology, Faculty of Medicine, Semmelweis University, 37-47 Tűzoltó Street, 1094 Budapest, Hungary; nadasy.gyorgy.laszlo@semmelweis.hu; 2 Department of Morphology and Physiology, Faculty of Health Sciences, Semmelweis University, 17 Vas Street, 1088 Budapest, Hungary; 3 Institute of Clinical Pathophysiology, Faculty of Medicine, Semmelweis University, 4. Nagyvarad Square, 1089 Budapest, Hungary; dezsi.laszlo@semmelweis.hu

Angiotensin II (Ang II) is essentially one simple peptide chain consisting of eight amino acids that spontaneously forms a steric structure with specific affinity to its pharmacological receptor. Despite the fact that it appears to be one of the simplest molecules in a world of peptides and proteins with as many as several hundred amino acids in their chains, it is present and has complex functions everywhere in the animal kingdom. It exists in an almost identical form from lower invertebrates to mammals and humans, with only one or two amino acid differences [1]. Angiotensin II was originally described almost a century ago as a sequential product of renin, the specific peptidase enzyme of the kidney, cleaving a decapeptide from the angiotensinogen protein, followed by the dipeptidase action of angiotensin-converting enzyme (ACE), resulting in the biologically active octapeptide. However, angiotensin II has been the source of many surprises for both scientists and clinicians, thanks to its complex activity [2]. While its originally identified main functions—controlling salt metabolism and body fluid pressure—are always present, it has developed and retained several additional functions that may historically have been useful but are potentially much less useful under current conditions. Complex production control, diverse possibilities for degradation, the potential presence of different receptor molecules in different tissues, different intracellular signal pathways with both heterotrimeric G protein-coupled receptor and cytokine receptor characteristics, endocytosis, recycling to the membrane, and intracellular processing of the receptor molecule all make the situation much more complex where angiotensin is involved in normal, extreme, and pathological conditions [3,4,5,6,7].

The significance of angiotensin II for global health cannot be overestimated. Several hundred million patients suffer from hypertension worldwide, with a large part of the global population consuming excessive quantities of salt, and an increasing percentage people taking antihypertensive drugs affecting the production and action of angiotensin II. Therefore, exploring the complexity of the renin–angiotensin system (RAS), with a focus on the angiotensin peptide, remains a current and future priority [1].

The pathological significance of angiotensin II is related on the one hand to the vulnerability of the complex salt-, body fluid- and vascular tone-controlling processes of which angiotensin II is an essential component. On the other hand, it has several additional effects on inflammatory, immunological, cellular metabolic, cell growth, cell differentiation, migration, apoptotic and even neural processes, which may not be ideal with regard to the salt and water-saving functions that may be required in complex new biological situations [1,7,8,9,10,11,12]. Angiotensin II has been proven to play an important role in tissue aging processes [13,14], and it should be seriously considered that it may even play a central role in biological longevity [15].

A biological approach is the key to understanding the complex functions of this simple peptide. Ten recent papers in this area have been collected in this Special Issue. Let us provide a brief introduction to each of them.

Ang II induces and exacerbates myocardial fibrosis in diabetes not only indirectly, through its vascular and hypertensive effects, but also directly by affecting the movement of calcium in the myocardial cells and promoting fibrotic transformation. This has been described in relation to experimental diabetic left ventricular damage in a complex in vivo–in vitro study by Paulik et al. [16].

Both ACE inhibitors (ACE-I) and angiotensin receptor blockers (ARBs) have significant immunomodulatory effects. ACE-I is a dipeptidase with low substrate specificity, affecting substrates other than Ang-I, such as some cytokines. The immunosuppressive effects of ARBs are explained by the partial molecular homology of the angiotensin type 1 receptor (AT1R) molecule with cytokine receptors, as well as the intracellular pathways they share with inflammatory transmitters. The application of such substances to treat autoimmune diseases, to suppress tumor growth and angiogenesis, and to reduce cognitive impairment in neurodegenerative diseases has been reviewed by Haliga et al. [17].

As described above, the diverse functions of the RAS can cause unexpected issues. Increased numbers of adverse cardiovascular effects have been reported in connection with Bruton’s tyrosine kinase inhibitor Ibrutinib, an approved drug for B cell malignancies that has demonstrated substantial survival benefits in chronic lymphocytic leukemia. In a clinical study by Font et al. it was found that elevated renin and angiotensin levels were responsible—at least in part—for these adverse cardiovascular events, limiting the clinical applicability of this otherwise useful drug [18]. From a scientific point of view, we hope that this interesting observation will motivate further studies revealing previously unobserved mechanisms controlling renin production.

Measuring ACE activity in urine may constitute an important clinical biomarker for evaluating kidney inflammation and tubular damage, as well as the activation of local RAAS-related pathways. However, such measurements are hampered by the presence of endogenous ACE inhibitors. A new laboratory technique has been developed to overcome this problem. Further studies on these endogenous ACE inhibitors will reveal new pathological mechanisms of kidney damage, as described by Szabo et al. [19].

Both hypertension and diabetes are common diseases, and the number of patients suffering from both conditions globally is increasing. Taking into consideration the complex activity of angiotensin, an interesting question is raised. How do commonly applied antihypertensive drugs, which inhibit the renin–angiotensin–aldosterone system (RAAS), interfere with popular antidiabetic sodium–glucose cotransporter type 2 (SGLT2) inhibitors? This question is reviewed in detail by Aazar et al. [20]. Their conclusion is favorable: double inhibition has both cardioprotective and renal protective effects.

A new, simple, biochemical interconnection between blood pressure and metabolic control mechanisms has been described by Enyedi et al. [21]. Blood plasma albumin levels, along with their attached fatty acids, have been shown to modulate the activity of ACE. The authors suggest considering such effects when evaluating the actions of ACE inhibitors.

The vasodilatory effect of the endogenous angiotensin II analog Ang (1–7), an ACE2 product, has now been demonstrated by Daci et al. [22] in both veins and arteries. Similarly to in arteries, this agonist is produced by ACE2 activity, which moderates the vasoconstrictor effect of angiotensin II in veins. This may be an important observation for phlebologists, who manage the venous conditions of patients with hypertension and treat them with RAS inhibitors. The cellular mechanism of this vasodilatory effect has also been revealed: Ang (1–7) exerted a substantial vasodilatory effect on rat vena cava inferior smooth muscle cells through activation of the Mas receptor and tetraethylammonium (TEA)-sensitive potassium channels.

The complex machinery of the intracellular signaling pathway of the angiotensin type 1 receptor provides opportunities to synthesize peptide analogs that have different effects on the heterotrimeric G protein-coupled and the c-Jun N-terminal kinase (JNK, pro-apoptotic) downstream intracellular pathways. Seven new angiotensin peptide analogs have been synthetized and tested by Matsuo et al. [23], with promising results. The identification of these so-called “biased” agonists is a promising area for future drug development. Significant theoretical results may also be obtained via analysis of the steric structure of their connections with the extracellular part of the AT1R molecule and identification of the subsequent steric molecular movements. This may lead to a description of the mechanical molecular lever system transmitting the signal through the phospholipid membrane toward the intracellular loops of the receptor.

The role of the RAAS in hypertensive emergencies has been reviewed by Minari et al. [24]. Their conclusion is that the derailment of the RAAS function is a critical component in the development of organ damage in such fatal situations. Targeted therapy should therefore be an important aspect of treatment.

Two of the main biological roots of the hypertension pandemics in recent times (in addition to urbanization stress, hedonistic food intake, and general aging of the population) are physical inactivity and a higher than optimal level of activity in the RAAS. The potential interaction between these two factors is analyzed by Oliveira et al. [25].

This Special Issue thus provides the latest insights into the complexity of the cell signaling and biological mechanisms of the RAS and proposes novel mechanisms in cardiovascular physiology and pathophysiology. By targeting this system, the presented works may provide novel therapeutic potential in cardiovascular and related diseases such as hypertension, heart failure, atherosclerosis, and diabetes mellitus.

## Data Availability

No new data were created or analyzed in this study. Data sharing is not applicable to this article.

## Abbreviations

The following abbreviations are used in this manuscript:

ACE	angiotensin-converting enzyme
ACE2	angiotensin-converting enzyme type 2
ACE-I	angiotensin-converting enzyme inhibitor
Ang II	angiotensin II
Ang (1–7)	angiotensin 1–7
ARB	type 1 angiotensin receptor blocker
AT1R	type 1 angiotensin receptor
JNK	c-Jun N-terminal kinase
RAS	renin–angiotensin system
RAAS	renin–angiotensin–aldosterone system
SGLT2	sodium–glucose cotransporter type 2
TEA	tetraethylammonium
